# De novo transcriptome reconstruction and annotation of the Egyptian rousette bat

**DOI:** 10.1186/s12864-015-2124-x

**Published:** 2015-12-07

**Authors:** Albert K. Lee, Kirsten A. Kulcsar, Oliver Elliott, Hossein Khiabanian, Elyse R. Nagle, Megan E.B. Jones, Brian R. Amman, Mariano Sanchez-Lockhart, Jonathan S. Towner, Gustavo Palacios, Raul Rabadan

**Affiliations:** Department of Biomedical Informatics, Columbia University College of Physicians and Surgeons, 1130 St. Nicholas Ave, New York, USA; Department of Systems Biology, Columbia University College of Physicians and Surgeons, 1130 St. Nicholas Ave, New York, USA; United States Army Medical Research Institute for Infectious Disease, Center for Genome Sciences, 1425 Porter St, Ft Detrick, 21702 USA; Centers for Disease Control and Prevention, Viral Special Pathogens Branch, 1600 Clifton Rd. NE, Atlanta, 30333 USA; National Center for Biodefense and Infectious Disease, George Mason University, Manassas, 20110 USA

**Keywords:** RNA-seq, Transcriptome, Genomics, Annotation, Database

## Abstract

**Background:**

The Egyptian Rousette bat (*Rousettus aegyptiacus*), a common fruit bat species found throughout Africa and the Middle East, was recently identified as a natural reservoir host of Marburg virus. With Ebola virus, Marburg virus is a member of the family *Filoviridae* that causes severe hemorrhagic fever disease in humans and nonhuman primates, but results in little to no pathological consequences in bats. Understanding host-pathogen interactions within reservoir host species and how it differs from hosts that experience severe disease is an important aspect of evaluating viral pathogenesis and developing novel therapeutics and methods of prevention.

**Results:**

Progress in studying bat reservoir host responses to virus infection is hampered by the lack of host-specific reagents required for immunological studies. In order to establish a basis for the design of reagents, we sequenced, assembled, and annotated the *R. aegyptiacus* transcriptome. We performed *de novo* transcriptome assembly using deep RNA sequencing data from 11 distinct tissues from one male and one female bat. We observed high similarity between this transcriptome and those available from other bat species. Gene expression analysis demonstrated clustering of expression profiles by tissue, where we also identified enrichment of tissue-specific gene ontology terms. In addition, we identified and experimentally validated the expression of novel coding transcripts that may be specific to this species.

**Conclusion:**

We comprehensively characterized the *R. aegyptiacus* transcriptome *de novo*. This transcriptome will be an important resource for understanding bat immunology, physiology, disease pathogenesis, and virus transmission.

**Electronic supplementary material:**

The online version of this article (doi:10.1186/s12864-015-2124-x) contains supplementary material, which is available to authorized users.

## Background

Bats (order: Chiroptera) constitute an abundant and diverse mammalian lineage comprising approximately 20 % of all known mammalian diversity [1]. Bats have evolved apart from other mammals for more than 50 million years [2] and are divided into two major suborders; the Yinpterochiroptera (megachiroptera) and the Yangochiroptera (microchiroptera). Yinpterochiroptera includes the family Pteropodidae and genera *Rousettes* and *Pteropus* whereas Yangochiroptera includes the family Myotidae and genus *Myotis* [3]. Unlike most mammals, bats can fly and this ability enabled their wide geographical range and increased metabolism [2]. Interestingly, bats have recently come to the forefront of zoonotic disease research with vast number of pathogens identified in a wide variety of bat species [2].

Upwards of 85 different viruses, primarily RNA viruses, have been detected and/or isolated from bats [2, 4]. Amongst these are emerging viruses that cause lethal disease in humans and nonhuman primates including Nipah virus [5, 6], Hendra virus [7], severe acute respiratory syndrome (SARS)-like coronavirus [8], Middle East respiratory syndrome coronavirus (MERS-CoV) [9], Marburg virus (MARV) [10–13], and Ebola virus (EBOV) [14–16]. Despite the severe virulence of these viruses in humans, infected bats are often asymptomatic [13, 17–22]. Nipah virus and Hendra virus interactions with their natural reservoir hosts, *Pteropus vampyrus* and *Pteropus alecto*, respectively, are well characterized. Experimental infections of bats with high doses of henipaviruses have shown virus replication and shedding with little to no disease [20–22]. Remarkably, the only viruses known to have induced any observable pathology in bats are rabies virus and Australian bat lyssavirus [2, 23]. Understanding mechanisms of disease and differential responses to infection in asymptomatic reservoir host species compared to species that exhibit severe pathology will help inform the development of novel therapeutics and disease prevention approaches.

*Rousettus aegyptiacus*, commonly known as the Egyptian rousette bat, has been identified as a natural reservoir host for MARV through ecological, epidemiological, and experimental studies [10, 12, 13, 18, 19, 24]. Furthermore, it has been speculated this bat could host Ebola virus [12, 25–27], although recent experimental infection studies have shown Ebola virus does not replicate well in *R. aegeyptiacus* [28]. The majority of human outbreaks due to MARV have been associated with caves inhabited by *R. aegyptiacus*. Furthermore, epidemiological surveillance of the *R. aegyptiacus* colony located in the Python cave in Uganda revealed a biannual spike in Marburg virus prevalence. This pattern correlated strongly with spillover transmission events in humans [24]. Initial studies in captive bats evaluated clinical signs, virus dissemination, and virus shedding patterns during experimental infection with a MARV isolate derived from wild bats [13]. Consistent with a natural reservoir host, the bats showed little to no evidence of disease even though the virus disseminated throughout their body and was actively shed [13]. These results were confirmed when bats were infected with MARV Angola, a strain isolated from a lethal human case [18]. In the absence of genetic and transcriptomic information for *R. aegyptiacus* and with limited available reagents, studying this reservoir host animal model has been challenging.

The rapid expansion in genomic knowledge for different bat species has facilitated comparative studies that rely on the identification of genes and gene families, and has established a framework for developing necessary reagents. Full genome annotations for *Pteropus vampyrus* (2.63X, [29]), *Myotis lucifugus* (6.6X, [29]) *Pteropus alecto* (110x, [30]), *Myotis davidii* (110x, [30]), and *Myotis brandtii* (77.8X, [31]) are now available. Additionally, transcriptomic annotations for *Pteropus alecto* [32] and *Artibeus jamaicensis* [33] have been published. In particular, the complementary genome and transcriptome annotations for *P. alecto* has aided studies on henipavirus infections in its reservoir host [30, 32]. The host transcriptional response to different viruses was also recently assessed in a kidney cell line derived from *P. vampyrus* utilizing the previously annotated genome [34].

In this manuscript, we report the transcriptomic annotation of *R. aegyptiacus* from a *de novo* assembly of RNA sequencing data from 11 tissues isolated from a male and a female bat. We identified 24,118 canonical coding transcripts whose expression profiles were consistent with the corresponding tissues of origin. In addition, we identified and validated novel coding transcripts that do not have any homology with the known sequences. Furthermore, we evaluated the annotation for immune-related genes and assessed the presence and expression of genes associated with a variety of immune functions.

## Results and discussion

### *De novo* transcriptome assembly of *R. aegyptiacus*

**Fig. 1:**
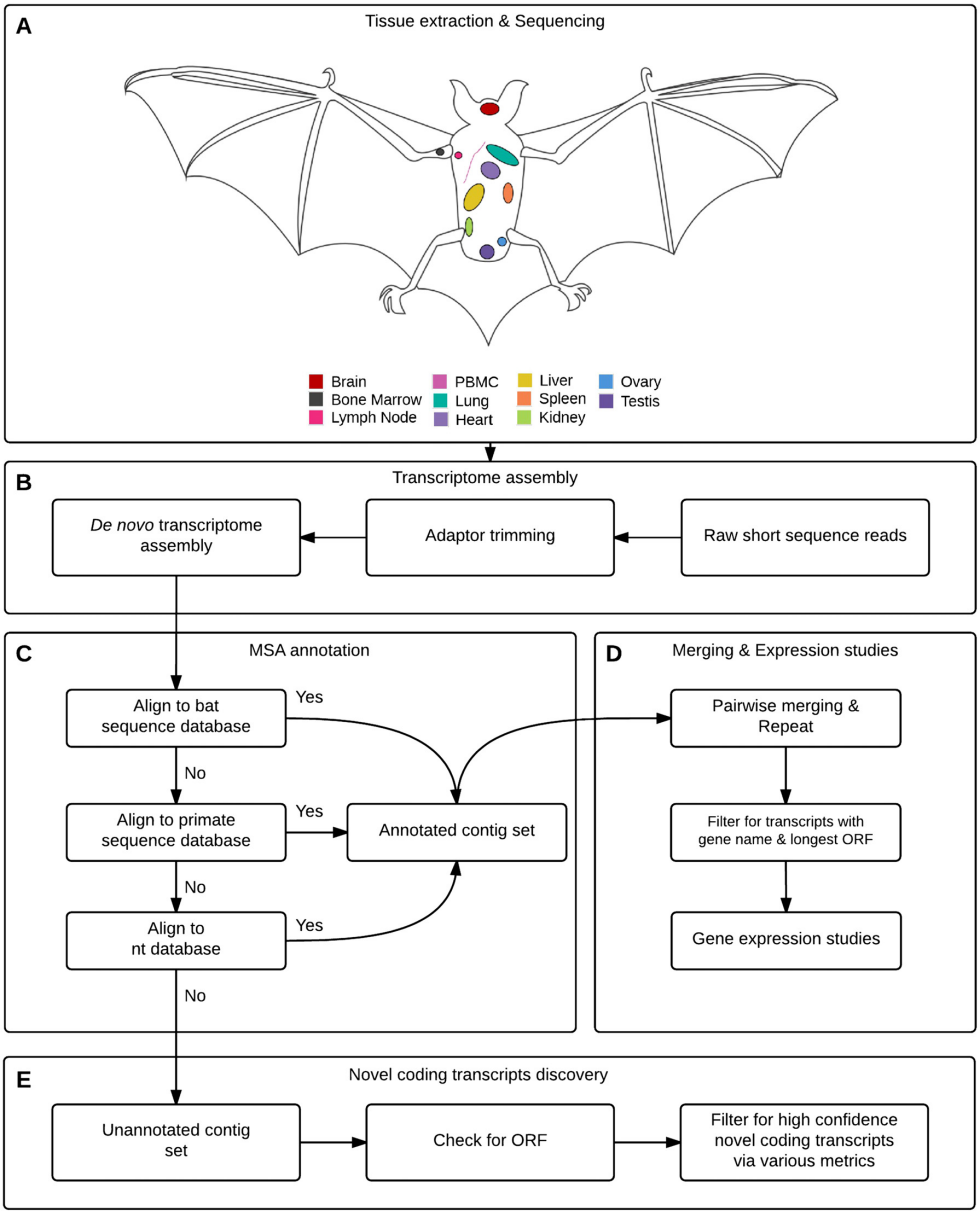
Schematic of the *de novo* transcriptome reconstruction and analysis pipeline. The pipeline consists of 5 steps. **a** Data generation: Multiple tissues are extracted from *R. aegyptiacus* and sequenced. **b** *De novo* Transcriptome assembly: Individual samples are first preprocessed to remove adapter sequences and assembled into contigs *de novo*. **c** MSA annotation: Once the set of contigs is generated, they are annotated using BLAST against three databases. In each step, unannotated contigs are iteratively annotated using the downstream databases. **d** Mering and Expression studies: A nonredundant contig set is obtained by merging the contig set of individual tissues two at a time. This pairwise merging is repeated until only one contig set is left. The subset of this contig can be obtained for the downstream analysis such as gene expression analysis by taking the transcripts with gene symbol and ORF sequence. See Fig. 2 for details. **e** Discovery of Novel Coding Transcripts: Novel coding transcripts can be identified by searching for contigs that failed annotation in the previous steps. Various metrics can be applied to generate high confidence novel coding transcript candidates

We employed a *de novo* assembly approach to generate a comprehensive transcriptome without relying on a genome reference. First, we generated 20 RNA-seq libraries consisting of 11 tissue types (Table 1, Fig. 1a) each collected from one male and one female *R. aegyptiacus* bat, which yielded approximately 2.1 billion reads. We then assembled the high quality reads using Trinity [35] (Fig. 1b). This process generated 14,796,219 contigs. The assembly had high continuity and coverage with a median number of 718,807 contigs and median N50 of 1,540 across all tissues (Table 1). To comprehensively annotate the contigs, we used the Multiple Species Annotation (MSA) pipeline [36], which leverages the homology of known sequences of related species. We assigned gene symbols to contigs when this information was available. This process clustered the contigs into isoform groups (Fig. 1c).

**Table 1:** Library Information and Assembly Statistics

Bat	Gender	Tissue	Read count	Library	N50	Number of contigs
BAT01	F	BM	67896687	single	1736	609943
BAT02	F	BR	55004118	single	884	896445
BAT03	F	HT	77315750	single	1263	717588
BAT04	F	KY	59782352	single	1174	720026
BAT05	F	LG	77510852	paired	1822	903831
BAT06	F	LN	63170354	single	1566	638083
BAT07	F	LV	89970603	paired	1566	697125
BAT08	F	OV	75051316	single	1401	875888
BAT09	F	PB	56553369	single	1890	404332
BAT10	F	SP	56141808	single	1340	716771
BAT11	M	BM	47988156	paired	1808	744115
BAT12	M	BR	75378417	paired	1490	1088331
BAT13	M	HT	20042200	paired	748	497729
BAT14	M	KY	71478010	paired	1514	872829
BAT15	M	LG	15525010	paired	668	575991
BAT16	M	LN	88471565	paired	2186	797125
BAT17	M	LV	27358079	paired	925	431513
BAT18	M	PB	92707184	paired	1745	556053
BAT19	M	SP	98465277	paired	2141	873259
BAT20	M	TT	96476242	paired	1866	1179242

### *R.aegyptiacus* transcriptome captures a majority of bat transcripts

We compared our assembly to the transcriptomes of three related bat species -- *M. davidii*, *P. alecto*, and *M. brandtii*. Using BLAST, we recovered 90.1 % of *M. davidii* transcripts, 89.54 % of *M. brandtii* transcripts, and 97.38 % of *P. alecto* transcripts. This result is consistent with the evolutionary history of these bats considering that *P. alecto* and *R. aegyptiacus* belong to the same family of *Pteropodidae*.

### Combining the transcriptome to generate nonredundant contigs

**Fig. 2:**
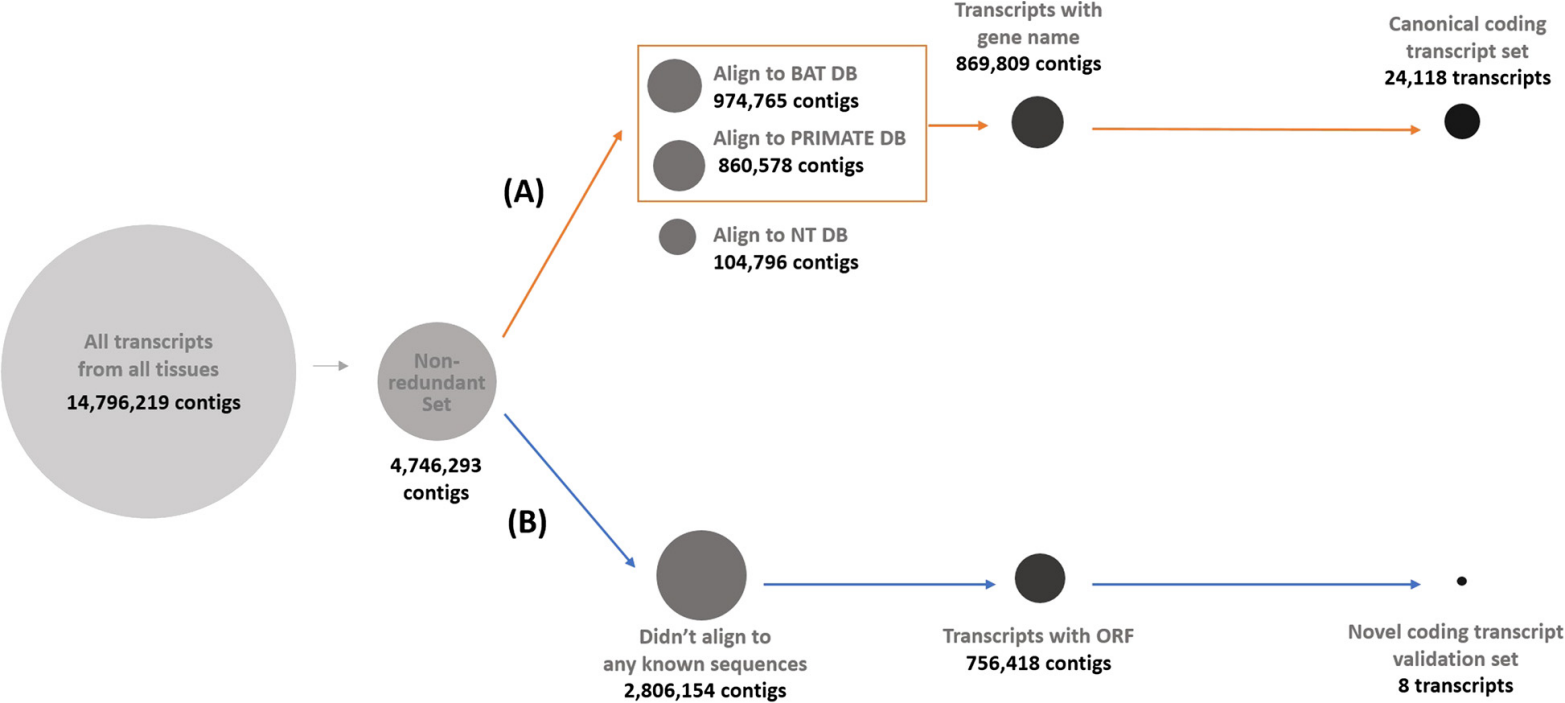
Generation of Nonredundant Contig Set, Canonical Coding Transcript Set, and High Confidence Novel Transcript Set. From the union of all contigs, we generated the nonredundant set of transcripts by iterative pairwise merging of contig set of all tissues; this yielded 68 % reduction of the contig set. **a** To generate Canonical Coding Transcript Set, we selected the contigs that are annotated with MSA pipeline. The annotated contigs are further filtered for contigs that have a gene symbol. For an individual gene cluster, we chose a transcript with the longest ORF to represent the corresponding gene (Canonical Coding Transcript Set). **b** For unannoated contigs, we selected for expression level, presence of an ORF with both start and stop codons in the CDS, and a minimum length of 400 nucleotides. We identified 8 high-confidence novel coding transcript candidates for validation

Tissue-specific transcriptome assemblies contained different numbers of contigs, due to their different levels of expression and sequencing depth. Without a common ground for comparison, it was difficult to perform downstream comparative analyses such as differential gene expression analysis; therefore, we combined contigs from all tissues into one unified, nonredundant reference transcriptome (Fig. 1d). To this end, we iteratively merged the assemblies two at a time, similar to the approach employed in [37] (Fig. 1d). We obtained 4,746,293 nonredundant contigs. Among the nonredundant contigs, 974,765 (20.54 %) of the sequences were annotated by bat sequences, 860,578 (18.13 %) by primate sequences, and 104,796 (2.2 %) by sequences in nt database (Fig. 2a). The nonredundant contig set had slightly lower sensitivity, though it still remained high; 86.60 % of *M. davidii*, 85.95 % of *M. brandtii*, and 95.30 % of *P. alecto* transcripts were recovered. The resulting annotated contigs were assigned gene names and combined using the longest annotated ORF as the transcript. This resulted in an annotation for *R. aegyptiacus* that contained a total of 24,118 genes. To determine the efficiency of using the MSA pipeline, we determined that 84 % (20,207 genes) of the contigs were annotated using the bat database and 16 % (3,911 genes) were subsequently annotated using the primate database. These data show that the MSA pipeline, which utilizes known transcripts from related species only, is a sensitive and efficient method for *de novo* transcriptome annotation.

### Biological validity via expression analysis

**Fig. 3:**
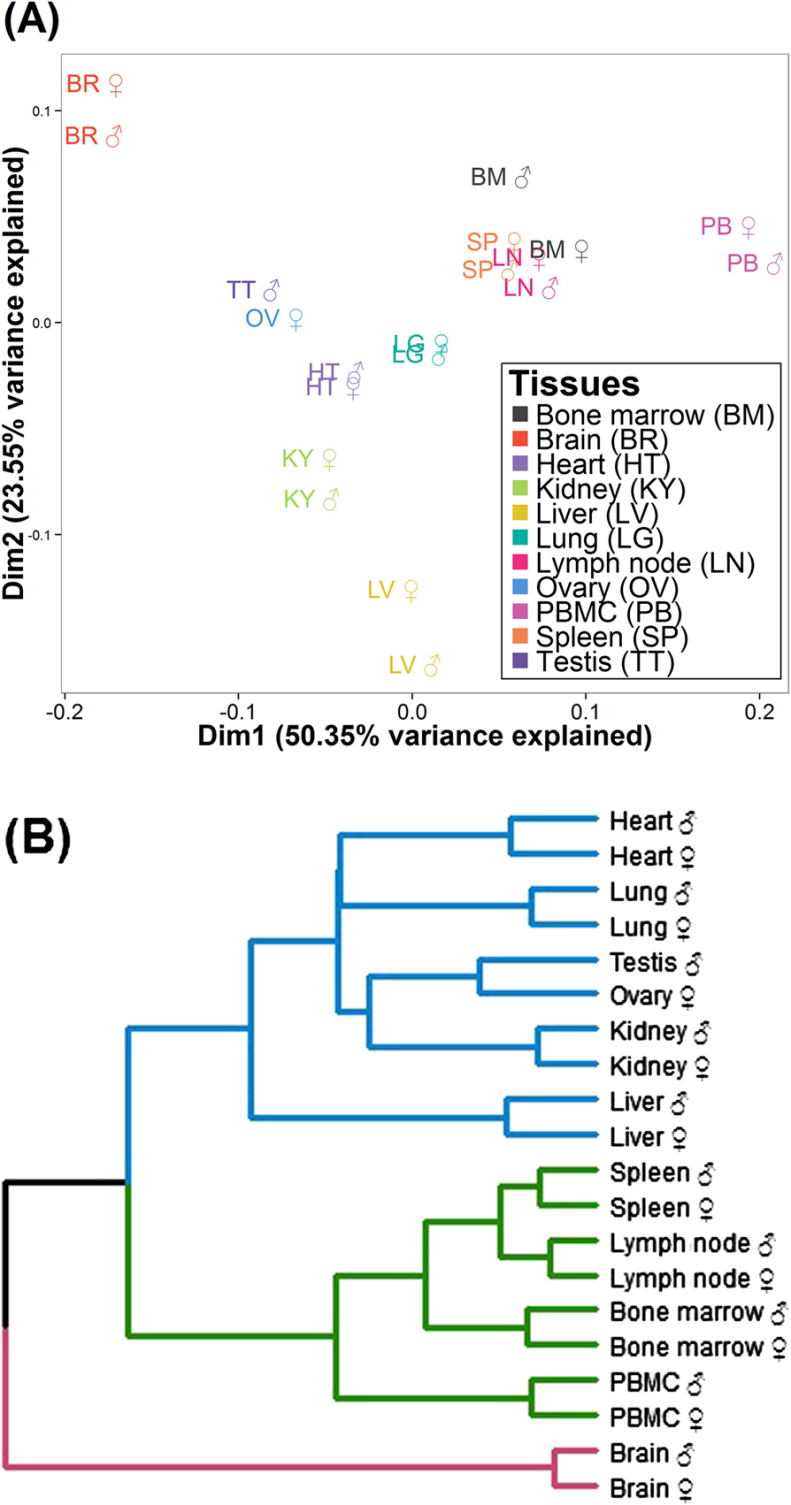
MDS of Gene Expression Profiles of Bat Tissues. **a** We assessed the biological validity and quality of our transcriptome annotations by performing Multidimensional Scaling (using 1-spearman correlation as distance) on gene expression profiles of all tissues using the 22,398 genes as feature vector. The first two coordinates explained 73.9 % of the variance in the data. **b** We performed hierarchical clustering of expression profiles using 1-spearman correlation as distance. The clustering suggested presence of three groups that correspond to separate developmental origins. Tissues used are Bone (BM), Brain (BR), Heart (HT), Kidney (KY), Liver (LV), Lung (LG), Lymph (LN), Ovary (OV), PBMC (PB), Spleen (SP), Testes (TT) of the male (M) and female (F) bat

We evaluated biological validity of the reconstructed transcriptome by analyzing global expression patterns across the different tissues. If the transcriptome assembly and annotations were accurate, the expression profiles of a given tissue should cluster with those of the same tissue origin and segregate from those of different origins [36, 38]. A gene can result in more than one transcript isoform; therefore, to capture the highest amount of information, for each gene, we focused on the transcript with the longest open reading frame (ORF) (Fig. 2a). After normalizing the expression values, we performed Multidimensional Scaling (MDS) to determine the relationships between the gene expression patterns in different tissues. As expected, MDS showed a clear separation of the samples according to the tissue of origin (Fig. 3a) and explains 74 % of the variance in the data. To examine the evolutionary relationship among tissues, we performed hierarchical clustering of the gene expression profiles (Fig. 3b). The brain, which has a different developmental pathway compared to the other organs, was classified as an outgroup. The spleen, lymph node, and bone marrow are all organs of the immune system and, as expected, clustered near each other. The peripheral blood contains some of the same cell types as the immune organs, thus, clustered near these tissues. Lastly, the gonads and kidney, which develop from the intermediate mesoderm, were grouped as neighbors in the tree. These results suggest that our transcriptome captured sufficient heterogeneity of gene expression to distinguish individual tissues while preserving their developmental relationships.

### Gene Ontology analysis

**Fig. 4:**
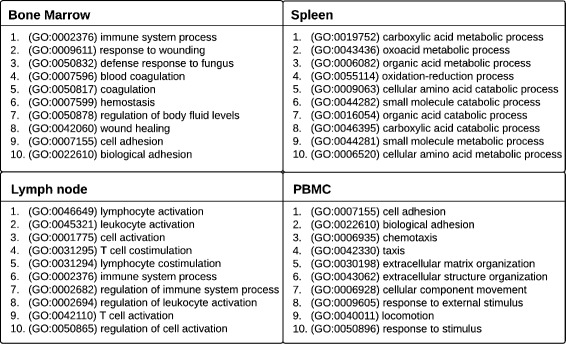
Top Ten Enriched Gene Ontology Biological Process Terms for bone marrow, spleen, lymph node, and PBMC. In each panel, the terms are listed in descending order of significance of enrichment. These tissues, in particular are associated with different aspects of the immune system and these associations are observed within the GO BP terms identified

We further assessed biological validity of our transcriptome assembly through gene Ontology (GO) analysis of tissue-specific expression profiles. We compared expression profile of each tissue with the average expression in the whole dataset, and identified the top 200 most differentially expressed genes based on a generalized linear modeling framework. Using this list, we examined the enriched GO biological process (BP) terms. Figure 4 shows the top 10 GO BP terms from the bone marrow, spleen, lymph nodes, and peripheral blood mononuclear cells (PBMCs). (For other tissues, see Additional file 1). Terms enriched for each tissue are consistent with their expected physiological functions.

### Identification of immune-related transcripts

**Fig. 5:**
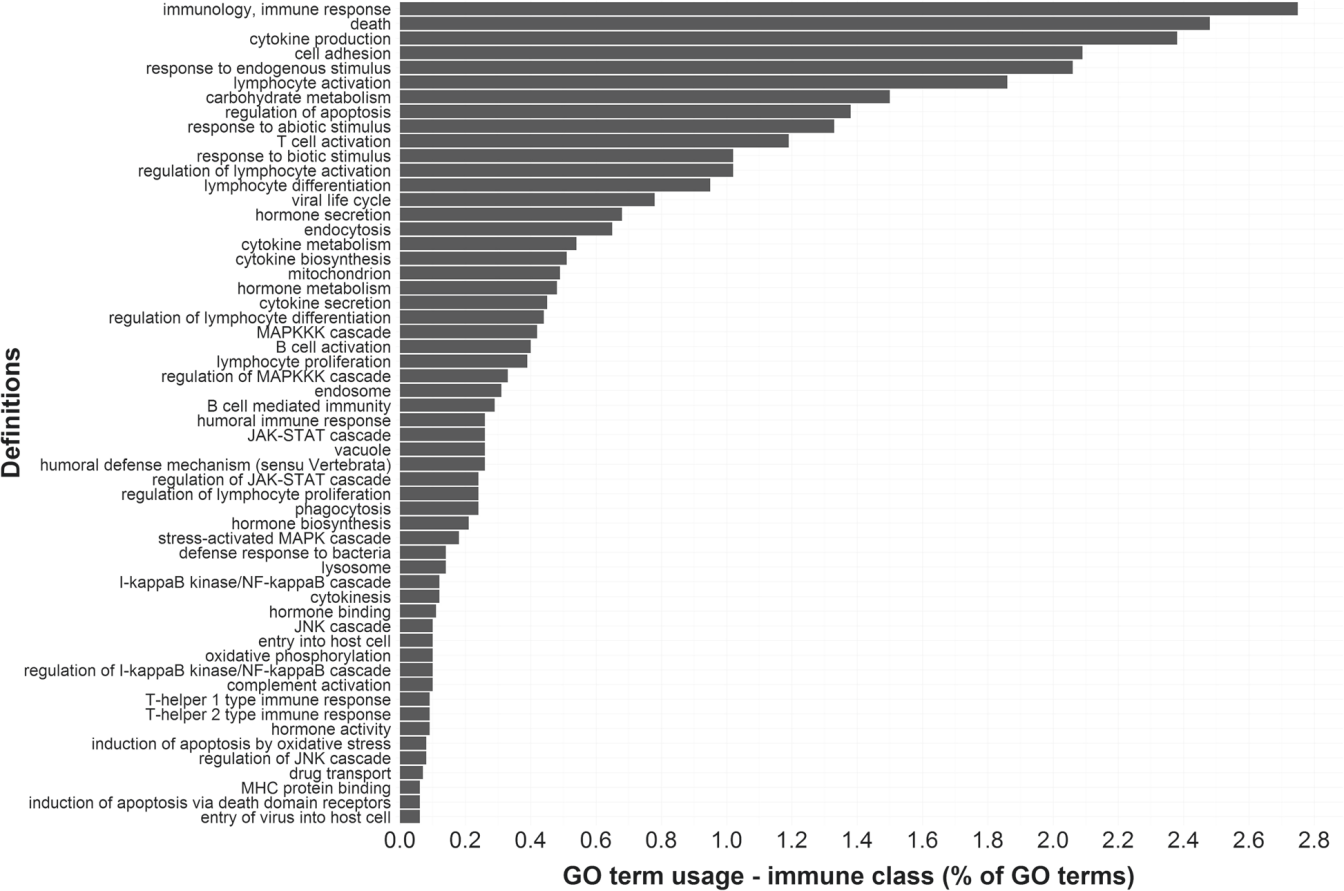
Distribution of immune genes within the *R. aegyptiacus* transcriptome at the GO Slim level using CateGOrizer. Genes annotated in the transcriptome were assessed for association with the immune response by analyzing them with CateGOrizer using the immune class GO Slim terms. The frequency shown is the percent of immune class GO slim terms associated with that particular pathway out of all the GO terms that were identified

*R. aegyptiacus* is a natural reservoir host for MARV, allowing for virus replication and dissemination with little to no pathological consequences [13, 17–22]. One important aspect of reservoir host biology is how their immune response compares to that of animal species that experiences severe disease, such as humans. Therefore, we examined the transcriptome for the presence of immune-related genes. We associated the R. aegyptiacus gene set with GO terms based on the human-specific gene ontology annotation. This resulted in 14,781 genes that mapped to 14,817 GO terms. We used CateGOrizer [39] and applied the immune class GOSlim terms to identify immune-related genes from this set. Similar to previous studies in *P. alecto* and *A. jamaicensis*, we found that out of 14,817 GO terms, approximately 2.75 % were associated with immune response [32, 33]. Amongst the most represented GO terms were cytokine production, lymphocyte activation, T cell activation, regulation of apoptosis, and regulation of lymphocyte activation (Fig. 5).

We next searched for specific genes related to various aspects of the immune response in other mammals, primarily mice and humans. We first evaluated the annotation of the transcriptome for the presence of anti-viral genes. A multitude of pattern recognition receptors were identified including toll-like receptors (TLRs) 1–9, RIG-I, MDA5, and LGP2 along with the important scaffold and signaling molecules Myd88 and MAVS. A variety of antiviral molecules were also found, including Mx1 and Mx2, PKR, STING, IRF3, IRF5, IRF7, members of the IFIT and IFITM families, and ISG15. We also looked for the presence of type I, II, and III interferons (IFN). We were able to identify IFN*gamma*, IFN*gamma*2, and IFN*alpha*. Transcripts corresponding to the IFN receptor subunits IFNAR1 and IFNAR2 were also identified. IFN*alpha* and IFN*beta* have been previously characterized by cloning from stimulated cells [40]. We, however, did not find any contigs corresponding to IFNB. To eliminate the possibility of an impaired assembly, we aligned the processed RNA-seq reads to the IFNB sequence from *P. alecto* [41] (Additional file 2 and Additional file 3). We detected only 2 reads from *R. aegyptiacus*,which did not provide sufficient coverage to construct the transcript. These data suggest that IFNB expression in healthy tissues of *R. aegyptiacus* is low, consistent with other mammals in which IFNB is primarily expressed after exposure to a stimulus.

We also searched the transcriptome for genes associated with innate immune cells. We found the transcripts for the CD14 and CD11c genes, which are commonly used for phenotyping macrophages and dendritic cells, as well as transcripts for the CD80 and CD86 genes, which are useful for evaluating the activation status of these cells. Genes associated with natural killer (NK) cells, however, were less evident. We were able to identify transcripts of co-receptor gene CD56, but not CD16. Transcripts of genes encoding for molecules in the killer cell lectin-like receptor (KLR) family, including NKG2A and NKG2D, were also not found. In other bat transcriptomes, such as *P. alecto* and *A. jamaicensis*, coverage of NK cell-related genes was more sparse than that of other mammals [32, 33]. A similar observation was made in the genome of *M. davidii* [30]. The absence of NK cell-related genes in the *R. aegyptiacus* transcriptome further strengthens the theory that bats might contain a different NK cell receptor repertoire than other species.

Next, we examined the repertoire of genes associated with adaptive the immune response. We identified a variety of transcripts associated with T cell identification, activation, inhibition, and differentiation including CD3 *ε*, CD4, CD8a, CD25, CD69, CCR7, PD-1, CTLA4, GATA3, foxp3, and Tbet. Interestingly, we were able to identify transcripts for the TCR *α* and TCR *β* chains, but were unable to find transcripts for the TCR *δ* and TCR *γ* chains. The transcriptome annotation for *P. alecto* included these genes, but they were present at low levels [32]. This supports the notion that *αβ* T cells are the predominant T cell subset in bats. We also looked at genes associated with B cells and were able to find transcripts for CD19, CD20, CD27, as well as transcripts that were similar to the immunoglobulin heavy chains A, E, G, and M and the immunoglobulin light chains *κ* and *λ*. Future analysis of the *R. aegyptiacus* genome is required to fully evaluate the immunoglobulin gene repertoire.

Finally, we studied the cytokine and chemokine repertoire, important for shaping both innate and adaptive immune responses. We found a variety of transcripts corresponding to a wide array of both pro-inflammatory and anti-inflammatory cytokines. These included IL-2, IL-4, IL-5, IL-6, IL-12a, IL-12b, IL-17a, IL-23, IL-10, TGF *β*, TNF, IFN *γ*, IL-1 *β*, CCL2, CCL5, and CXCL10. Altogether, the reference transcriptome generated for *R. aegyptiacus* provides an excellent foundation for investigating reservoir host immunology in bats.

### Novel transcripts

**Fig. 6:**
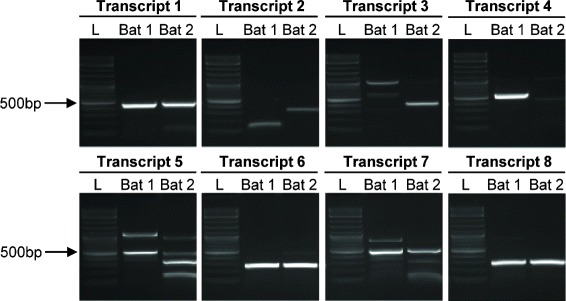
Unannotated, novel transcripts from *R. aegyptiacus* were validated of by RT-PCR. RNA from the spleen of both bats was reverse transcribed to make cDNA. The cDNA was amplified using primers specific for one of 8 novel transcripts that were unannotated in the assembly, but contained a complete ORF larger than 400 nucleotides. The expected product sizes were: transcript 1, 457 bp; transcript 2, 450 bp; transcript 3, 419 bp; transcript 4, 548 bp; transcript 5, 469 bp, transcript 6, 277 bp; transcript 7, 507 bp; and transcript 8, 301 bp

There were 2,806,154 unannotated contigs from the nonredundant contig set (Fig. 2b). Of those, 71.6 % (2,008,503 contigs) did not have an ORF suggesting the majority of these contigs may be noncoding transcripts. To determine if the unannotated contigs were real or artifacts from the assembly, we used BLAST to align this set of contigs to the *P. alecto* genome and found that 96 % (2,706,432 contigs) were aligned. To evaluate the possibility of an incomplete or impaired assembly, we grouped the aligned contigs into a total of 1,012,664 clusters based on the presence of overlapping sequences. This reduction suggests that multiple isoform expression patterns between different tissues may have affected our assembly or that our short read assembly may have been incomplete. Nonetheless, the number of unannotated contigs that aligned to the *P. alecto* genome suggests that these contigs, either coding or noncoding, may be novel transcripts shared within the order *Pteropodinae*. Future studies evaluating the conservation and possible functions of these sequences are essential to determine the importance of these genetic elements. To validate novel contigs in *R. aegyptiacus* that appeared to be coding we utilized PCR. Primers were designed to produce amplicons for eight highly expressed, unannotated contigs that contained ORFs longer than 400 bp. Using RNA isolated from the spleen, we were able to produce amplicons of the expected size from at least one bat (Fig. 6 and Additional file 4). The sequences of these amplicons were found to match the expected sequence from the assembled ORF of the unannotated contig. These contigs also showed high sequence similarity to the *P. alecto* genome. In particular, six of the 8 validated transcripts showed sequence similarity higher than 75 % at a query coverage greater than 64 %. The other two validated transcripts had a query coverage of 23 with 78.36 % identity (transcript 1 in Fig. 6) and a query coverage of 7 with 91.27 % identity (transcript 2 in Fig. 6) (Additional file 5); therefore, we hypothesize that these transcripts might be specific to *R. aegyptiacus*. Further investigation is needed to fully understand the characteristics and biological functions associated with the proteins these contigs encode.

## Conclusion

In this paper, we presented the comprehensively annotated of transcriptome of *R. aegyptiacus* and assessed its quality and biological validity. This transcriptome will be an important resource to study bat immunology. In particular, it will facilitate the process of investigating differences in host responses between asymptomatic reservoir host species and species that exhibit severe pathology. It will also pave the way for the development of novel therapeutics and prevention approaches against emerging zoonotic virus outbreaks.

## Methods

### Sample preparation

Tissues and blood were collected from one male and one female adult *R. aegyptiacus* bats that were bred and housed at the colony established at the Center for Disease Control and Prevention, Atlanta, GA, USA (Amman et al. 2015 [13]). Approximately 100 mg of the following tissues were collected and homogenized in 1 mL of Trizol LS (Invitrogen, Carlsbad, CA): liver (bat id:BAT7, BAT17), lung (BAT05, BAT15), heart (BAT03, BAT13), kidney (BAT04, BAT14), brain (BAT02, BAT12), axillary lymph nodes (bilateral, pooled) (BAT06, BAT16), spleen (BAT10, BAT19), bone marrow (BAT01, BAT11), and gonad (BAT08, BAT20). PBMCs (BAT08, BAT18) were isolated from the blood and stored in Trizol LS as well.

RNA was extracted using the PureLink RNA Mini kit (Invitrogen, Carlsbad, CA). cDNA was synthesized using the TruSeq Stranded Total RNA Sample Prep Kit (Illumina, San Deigo, CA) according to the manufacturer’s protocol. The libraries were evaluated for quality using the Agilent 2100 Bioanalyzer (Agilent, Santa Clara, CA). After quantification by real-time PCR with the KAPA qPCR Kit (Kapa Biosystems, Woburn, MA), libraries were diluted to 10 nM. Cluster amplification was performed on the Illumina cBot and libraries were sequenced on the Illumina HiSeq 2500. Eight of the female bat libraries were single-end, while the remaining tissues from the female bat and all tissues from the male bat were paired-end. All of the libraries sequenced were 125 bp in length. The average library depth was 66 M reads (minimum 16 M and maximum 98 M).

### Ethics statement

All experimental procedures were conducted with approval from the Centers for Disease Control and Prevention (CDC, Atlanta, GA, USA) Institutional Animal Care and Use Committee, and in strict accordance with the Guide for the Care and Use of Laboratory Animals (Committee for the Update of the Guide for the Care and Use of Laboratory Animals 2011). The CDC is an Association for Assessment and Accreditation of Laboratory Animal Care International fully accredited research facility. No human patient-derived clinical materials were used in these studies.

### *De novo* transcriptome assembly

We first examined the quality of the reads using FastQC v0.11.3 [42]. We also preprocessed the reads to remove the adapter sequence using cutadapt v1.5 [43]. We removed “AGATCGGAAGAGCACACGTCTGAACTCCAGTCAC” from the forward strand and “AGATCGGAA-GAGCGTCGTGTAGGGAAAGAGTGT-AGATCTCGG- TGGTCGCCGTATCATT” from the reverse strand. We performed strand-specific de novo transcriptome assembly using Trinity r20140413p1 [35] with the parameters: “–normalize_reads” and “–SS_lib_type FR”, along with its default parameters for all of our samples.

### Homology based annotation of the transcriptome

For annotation of contigs and clustering them into a gene model, we used Multiple Species Annotation pipeline, an nucleotide-based annotation approach that is more efficient and faster than BLASTX [36]. To make a BLAST [44] database for bats, we started with the complete “Nucleotide collection” (nt) database. We exported all accession numbers of the bat sequences at NCBI and made a subset database from nt using “blastdb_aliastool -db nt -dbtype nucl -gilistbats.sequence.gi.txt -title Bats -out Bats”. Using the same type of query, we also created a database for primates including humans due to their extraordinarily well-annotated transcriptomes, which will maximize the power of our annotation pipeline. We then used BLAST to iteratively align the contigs to the bat db, the primate db, and finally nt using a subtractive approach: what did not align to the bat db was aligned to the primate db, and what did not align to the primate db was aligned to nt.

### Sensitivity of *R.aegyptiacus* transcriptome

To assess the coverage of our transcriptome, we downloaded the *M. davidii*, *P. alecto*, and *M. brandtii* transcriptomes from NCBI Eukaryotic genomes annotations [41]. We generated a BLAST index out of union of all contigs from our samples, and aligned the three bat contigs to our BLAST databases. We chose the alignment with 70 % of sequence identity with maximum evalue of 1e-4.

### Nonredundant transcriptome assembly

To generate a nonredundant set of contigs, we iteratively merged individual assemblies using the the methods similar to the [37] employed to merge the kmers. Using CD-HIT-EST v4.6 [45] with sequence identity threshold of 0.99, we merged the first two pairs of contig sets (of sample *i* and sample *i*+1) upto the final sample *n*. After each iteration, we merged the resulting merged contig sets using a similar approach until only one contig set remained.

### Canonical coding transcript set

For the expression profiling, we generated a reference transcriptome consisting of transcripts each representing a gene model according to the following method: We first used TransDecoder (r20140413p1) [46] to find the ORF of all transcripts. Then, based on the MSA pipeline, we chose a transcript with gene symbols and the longest ORF in each gene cluster to capture the most information for downstream expression analysis. We did not consider the contigs mapped to nt database in this manuscript because obtaining feature files for all sequences as required by the MSA pipeline was computationally impractical, and a majority of the gene symbols (24,118) are captured in the bat and primate databases.

### Gene expression and gene ontology analysis

After a canonical transcript set was obtained, we used this as a transcriptome reference for expression analysis. We mapped the preprocessed reads to this reference using RSEM v1.2.19 [47] and obtained a gene-to-count matrix. We removed the transcripts with expression variance equal to zero or with low expression (count <=10). For MDS plot, we used the spearman correlaton as a distance measure and “cmdscale” from the “stats” package in R [48]. To explore the biological processes in each gene expression profile, we employed a one-to-all sample comparison using the EdgeR generalized linear model framework [49, 50]. For each tissue, we compared individual gene expression within the tissue versus the average expression of all other tissues. With each tissue having differently ranked gene lists, we then selected top 200 genes and ran gene ontology analysis using topGO [51] with human-specific gene ontology annotation [52].

### Analysis of unannotated transcripts and identification of novel transcripts and validation

We used BLAST [44] to align unannotated contigs to the genome of *P. alecto* with the evalue of 1e-4 and query coverage of 40 %. To cluster the aligned contigs into groups, we used bedtools [53] setting the distance threshold parameter at 0. For transcripts that did not align with any similarity to bat, primate, or nt BLAST databases, we applied a series of filters to select for the coding transcripts to be validated. We used the following criteria: an ORF that was complete with both a start and stop codon, an ORF that was at least 400 bp in size, and a transcript that was expressed (a read count >0). We further selected for the novel transcripts with usuable primers using primer-BLAST [54]. Using these criteria, the number of novel transcripts was narrowed down to a total of 8. The primers and expected amplicon size are listed in Additional file 4.

For validation, RNA was extracted from the spleen tissue of both the male and female bats using Trizol LS (Invitrogen, Carlsbad, CA). cDNA was synthesized from 2.5 *μ*g of RNA using the Superscript III First-strand Synthesis SuperMix (Invitrogen, Carlsbad, CA). Amplicons for each of the primer sets were generated using Phusion HotStart Flex DNA polymerase (New England BioLabs, Ipswitch, MA) and run on a 1.5 % agarose gel for visualization. The correct size amplicon was gel extracted, quantified, and Sanger sequenced on the Applied Biosystems 3730 ×1 DNA Analyzer.

## Additional files

Additional file 1
**Gene Ontology analysis of all tissues.** Three sheets contain enriched GO terms of Biological Process, Molecular Function, and Cellular Compartment in individual tissues. (XLS 31 kb)

Additional file 2
**Alignment of** ***R. aegyptiacus*** **reads to** ***P. alecto*** **transcripts.** The preprocessed reads are aligned to the interferon and immunoglobulin transcripts of *P. alecto* obtained from [41] and [32]. The sequences used are described in Annotation file 2. (JPEG 191 kb)

Additional file 3
**Sequences used in Additional file** **2** **.** Information on sequences used in Additional file 1 is described. (XLS 25 kb)

Additional file 4
**Novel transcripts information.** Various Information on 8 novel coding transcripts are provided including average expression value, transcript length, CDS length, ORF length, transcript sequence, cds sequence, ORF sequence, primers used, and expected amplicon sizes. (XLS 90 kb)

Additional file 5
**BLAST results of validated novel transcripts**. The table is the BLAST output of the validated novel transcripts mapped to *P. alecto* genome. (XLS r27 kb)
