# Supplementary figures and images for: De novo transcriptome reconstruction and annotation of the Egyptian rousette bat
Source: BMC Genomics. 2015 Dec 7;16:1033. doi: 10.1186/s12864-015-2124-x (PMC4672546; doi:10.1186/s12864-015-2124-x)

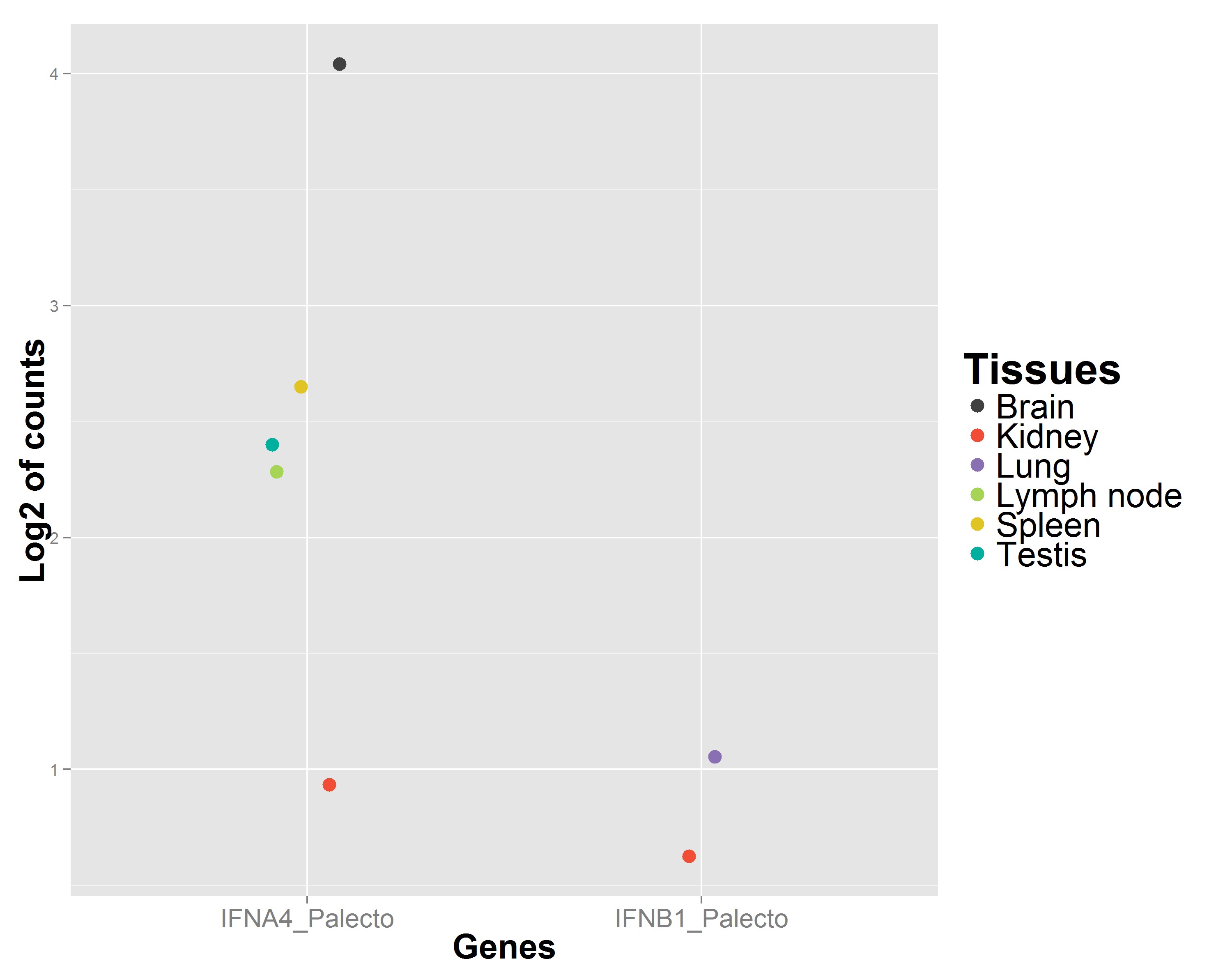

Supplement: Additional file 2 — Alignment of R. aegyptiacus reads to P. alecto transcripts. The preprocessed reads are aligned to the interferon and immunoglobulin transcripts of P. alecto obtained from [41] and [32]. The sequences used are described in Annotation file 2. (JPEG 191 kb) [file 12864_2015_2124_MOESM2_ESM.jpeg]
